# Treatment of Chronic Patellar Tendinopathy with Autologous Bone Marrow Stem Cells: A 5-Year-Followup

**DOI:** 10.1155/2012/953510

**Published:** 2011-12-18

**Authors:** Cecilia Pascual-Garrido, A. Rolón, A. Makino

**Affiliations:** ^1^Sports Medicine and Shoulder Surgery, Hospital for Special Surgery, 535 E 70th Street, New York, NY 10021, USA; ^2^Department of Radiology, Diagnostic Center Rossi, Arenales 2777, CABA, Buenos Aires 1425, Argentina; ^3^Knee Section, Center of Excellence Dr. Arturo Makino, Avenue, Las Heras 1947 9° Piso, CABA, Buenos Aires 1425, Argentina

## Abstract

The purpose of this study is to determine if patients with chronic patellar tendinopathy will improve clinically after the inoculation of bone marrow mononuclear cells (BM-MNCs). Eight patients with chronic patellar tendinopathy were included. Patients averaged 24 years old (range 14–35). All patients were refractory to conservative treatment for at least 6 months before the procedure. BM-MNCs were harvested from the iliac bone crest and inoculated under ultrasound guide in the patellar tendon lesion. Improvement was assessed through established clinical scores and ultrasound. At 5-year followup, statistically significant improvement was seen for most clinical scores. Seven of eight patients said they would have the procedure again if they had the same problem in the opposite knee and were completely satisfied with the procedure. Seven of 8 patients thought that the results of the procedure were excellent. According to our results, inoculation of BM-MNCs could be considered as a potential therapy for those patients with chronic patellar tendinopathy refractory to nonoperative treatments.

## 1. Introduction

Chronic patellar tendinopathy is a common and disable disease. Despite improvements in early detection, evaluation, and advance rehabilitation techniques, treatment is long and tedious. Multiple approaches, both surgical and nonsurgical, have been suggested.

The precise mechanism by which tendinopathy develops in humans is not quite understood. Histopathology studies have consistently shown that tendinopathy in humans is typically due to tendinosis [1]. Tendinosis is characterized histologically by tissue degeneration with failed reparative response and absence of inflammatory cells. The pathological region is distinct from normal tendon with both matrix and cellular changes. Instead of clearly defined, parallel, and slightly wavy collagen bundles, tendinosis is associated with relative expansion of the tendinous tissue, loss of the longitudinal alignment of collagen fibers, and loss of the clear demarcation between adjacent collagen bundles. Multiple cellular changes coexist with these matrix changes. The most obvious is hypercellularity resulting from an increase in cellular proliferation. There is atypical fibroblast and endothelial cellular proliferation and extensive neovascularization. It has been suggested that fibroblast in tendinosis had an abnormal respond to healing stimulus, probably due to cell transformation [2]. Increased expression of MMP has also been shown in diseased tendons [3].

Recent work *in vitro* has focused on the use of bone marrow mononuclear stem cells (BM-MNCs) combined with growth factors to improve the quality and speed of healing in tendinosis repair. Clinically, PRP or platelet-rich plasma has shown to improve pain and function over time in patients with chronic patellar tendinopathy [4–6].

BM-MNCs are pluripotential cells and are believed to play an important role in connective tissue repair such as tendon, ligament, bone, and cartilage. Several animal studies have shown that the inoculation of these cells can accelerate tendon healing [7–9]. A combination of BM-MNCs and anabolic growth factors would seem an attractive approach for improving connective tissue repair.

The objective of this study is to evaluate the outcomes of inoculation of BM-MNCs in patients with chronic patellar tendinopathy of the knee through established outcomes scales, clinical assessment followup, and ultrasound images.

## 2. Materials and Methods

### 2.1. Patient Selection

Between June 2005 and September 2006, 8 consecutive patients with chronic patellar tendinopathy treated with mononuclear BM-MNCs were included. Approval for the study was obtained by the institutional review board at our institution, and all patients signed informed consent to participate. Indications for BM-MNCs were history of pain (more than 6 months), tenderness on patellar tendon palpation, and imaging findings of degenerative changes (MRI). All patients had failed previous nonoperative treatment for at least six months including all or a combination of conventional stretching exercises and strengthening, and were classified as grade III-B according to the criteria originally described by Blanzina and later modified by Warden et al. [10] (patients unable to participate in sports at the same level as before the onset of symptoms). Exclusion criteria were systemic disorders such as diabetes, rheumatoid arthritis, coagulopathies, cardiovascular diseases, infections, immunodepression, patients in therapy with anticoagulants, and use of NSAIDs in the five days before bone marrow aspiration. All patients had an ultrasound (US) done before surgery and at 6 months postoperatively. Based on US findings, four grades of patellar tendinopathy were identified (Table 1) [11].

### 2.2. Bone Marrow Aspiration

Under general anesthesia, bone marrow was aspirated from anterior iliac crest using a bone marrow harvest needle (Medical Device Technologies, Inc., Gainesville, Fla, USA) with 20-mL syringes prefilled with anticoagulant citrate dextrose (1.5 mL) to prevent blood clotting. Ficoll-Paque Premium (3 mL) was added to centrifuge tubes. Then the bone marrow was carefully layered in the Ficoll-Paque Premium, making sure that the bone marrow sample did not mix with the Ficoll-Paque Premium. Then centrifugation was done, and the upper layer containing plasma and platelets using sterile pipette was drawn off leaving the mononuclear cells undisturbed at the interface. The layer of mononuclear cells was transferred to a sterile centrifuge tube. These cells were washed by centrifugation. Supernant was removed and the mononuclear cells were resuspend in 6 to 8 mL balanced solution appropriate for application.

### 2.3. Haematological Analysis

Haematological analysis was performed according to a previously published method [12]. The numbers of nucleated cells in BMA (bone marrow aspiration) were determined before and after concentration with an automated haematology analyzer. The concentration ratio was determined as follows: concentration ratio = number of nucleated cells in BMA after concentration/number of nucleated cells in BMA before concentration.

### 2.4. Inoculation

**Figure 1:**
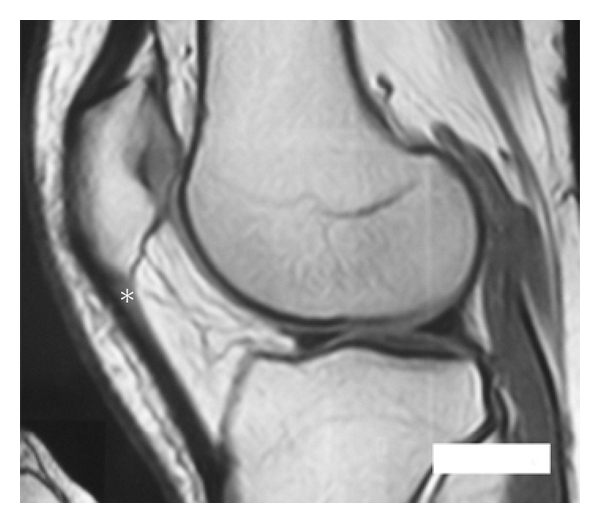
Preoperative magnetic resonance imaging showing hypodens area in the proximal aspect of the patellar tendon (*).

**Figure 2:**
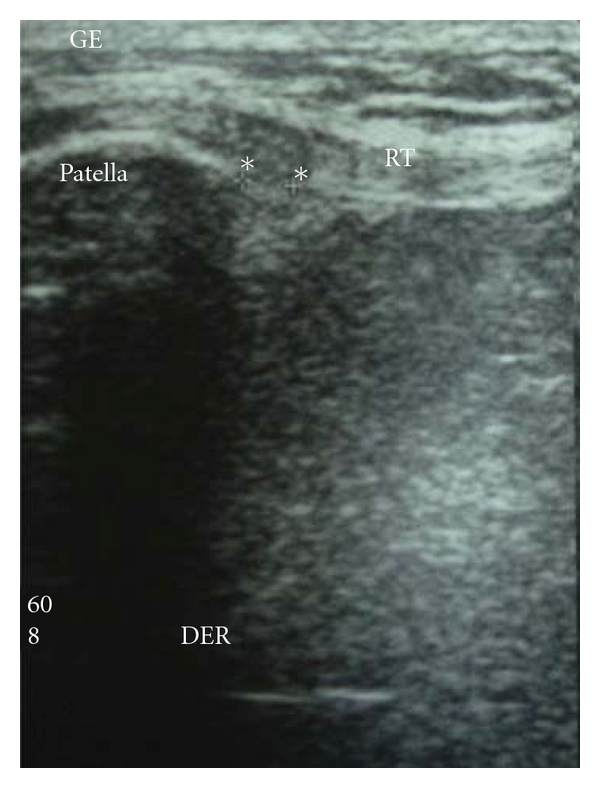
Preoperative US (ultrasound) of the patellar tendon showing areas of edema (hypoechoic) (**). RT: rotulian or patellar tendon.

**Figure 3:**
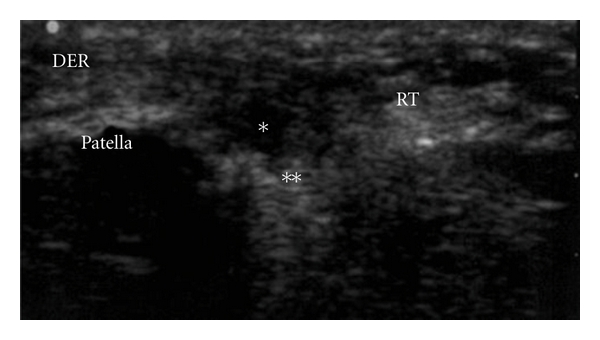
Under US guide, the cells were inoculated. Observe the hypoechoic area (*) where the cells were inoculated. An acustic shadow is evident after the inoculation of the cells (**). RT: rotulian or patellar tendon.

The area of injury was identified, and the zone was clearly marked based on physical examination, imaging studies, and area of maximal tenderness. An ultrasound-guided injection was used to better identify the pathologic area. (Figures 1, 2, and 3). After the injection, the patients were sent home with instructions to limit the use of the leg for at least 24 hs and use cold therapy for pain. Then, patients started with stretching exercises and mild activities (such as bicycle exercises and mild exercised in the pool). After a month, patients were allowed to do recreational sports or activities as tolerated.

### 2.5. Outcomes Assessment

Only patients with a minimum 24 months followup were included for analysis. Questionnaires were administered preoperatively, 1 year postoperatively and then annually. Subjective measures were based on several scoring systems including Lysholm, Tegner, Cincinnati, international knee documentation committee (IKDC), knee injury ad osteoarthritis outcome score (KOOS) and Short Form-12 (SF12) [13–15]. The KOOS holds 5 separately scored subscales: pain, other disease-specific symptoms, activities of daily living (ADL) function, sport and recreation function, and knee-related quality of life (QOL) [12]. Patients were also asked to rate the overall condition of their knee at the time of the last followup: 0 to 2 poor (significant limitations that affect activities of daily living); 3 to 4, fair (moderate limitations that affect activities of daily living, no sport possible); 5 to 6, good (some limitation with sports but I can participate, I compensate); 7 to 8, very good (rare limitations, able to participate); 9 to 10, excellent (able to do whatever I wish no problems). Patient satisfaction with the procedure was elicited with the following scale: completely satisfied, mostly satisfied, somewhat satisfied, and unsatisfied. Finally, the patients were asked if, based on their experience, they had the same problem in the opposite knee, would they have the same surgery again.

An ultrasound was performed in all patients prior to inoculation and at 6 months after the injection to objectively evaluate tendon healing.

### 2.6. Statistical Analysis

Descriptive statistics were calculated according to standard methods, including frequencies, means, standard deviations, and ranges when appropriate. Clinical outcome scores were analyzed at 2 time points: preoperatively and at the most recent followup. Score improvement was calculated using a paired t test. A statistical significance was set at *P* < 0.05. Statistics were performed using GraphPad software (GraphPad Software, La Jolla, Calif, USA).

## 3. Results

**Figure 4:**
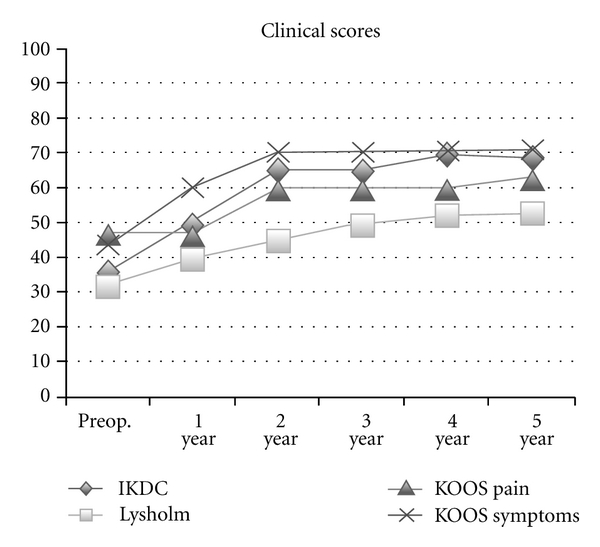
Preop. and yearly time follow-up for IKDC, Lysholm, KOOS pain and symptoms.

The mean age of the patients was 24 (range 14–35). Four patients were female and four male. No complications were recorded. The average total number of cells inoculated injected was 30 × 10^3^. The average patient followup was 5 years (range, 3–6). Overall, statistically significant improvement (preoperative to postoperative) for the Tegner (2 to 8, *P* = 0.006), IKDC scores (36 to 69, *P* = 0.047), KOOS symptoms (44 to 71 *P* = 0.0086), KOOS ADL (63 to 90, *P* = 0.0086), KOOS sport (24 to 63 *P* = 0.0078). No statistical improvement was seen for the Lysholm (33 to 53 *P* = 0.1043), KOOS pain (47 to 63 *P* = 0.2399), KOOS QOL (50 to 71 *P* = 0.0825), SF-12 mental (52 to 57 *P* = 0.5589) and SF-12 physical (41 to 44 *P* = 0.438). Detailed overall results are shown in Table 2. Considering each time followup, there was significant improvement at 2 years and plateau till last follow-up at 5 years (Figure 4).

Seven of eight patients said they would have the procedure again if they had the same problem in the opposite knee. Seven of eight patients were completely satisfied with the procedure, one patient was somewhat satisfied. Seven of 8 patients thought that the results of the procedure were excellent (10, scale from 0–10). None of the patients had additional procedures.

### 3.1. Cells Inoculated

The number of nucleated cells obtained from bone marrow aspiration was 37 × 10^3^ cells (±10). After concentration (cell recovery 85%) the average total numbers of BM-MNCs were 45 × 10^3^ (±5).

### 3.2. Ultrasound Evaluation

**Figure 5:**
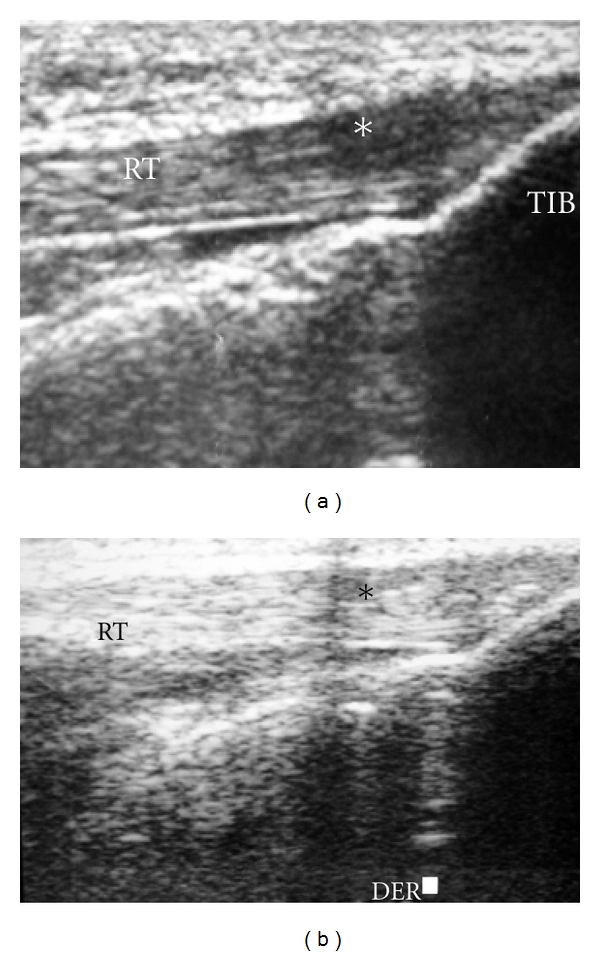


All patients were grade 2-3 before inoculation. At 6 months 8 of the nine patients had grade 1. Only one patient had grade 3 (Figure 5). 

## 4. Discussion

In this study, 8 patients with chronic patellar tendinopathy were treated with autologous BM-MNCs. We compared the outcomes of patients before and at 5 years after the inoculation, showing statistical improvement in most of the outcomes scores at the time of followup. Patients reached a plateau after one year followup. Although this can be argued as may be a self-limiting process, none of our patients had a recurrence which is normally reported to be between 12 to 27% in patients treated nonoperatively [9]

The major limitation of this study is the lack of control group, resulting in a low level of evidence study (Level 4) and the few number of patients that were included. Although we did not compare MRI pre- and postoperative to assess healing, we used ultrasound to assess healing before and at last followup. Warden et al. compared the accuracy between MRI and US in confirming clinical diagnosis of patellar tendinopathy. They suggested that US is more accurate than MRI confirming clinical diagnosed patellar tendinopathy (83% versus 70% resp.) [10]. To our knowledge, there have not been published articles that assess the effect of BM-MNCs for the treatment of patellar chronic tendinopathy in patients.

Treatment of chronic patellar tendinopathy is challenging secondary to the low capacity of healing that the tendon has resulting in long and tedious treatments. Identifying alternative strategies is a priority. The tendon itself is relatively cell poor, with a low turnover rate. Recently, it has been proposed that adult stem cells would be good candidates for cell-based tendon regeneration [9, 16]. The exact role of implanted stem cells on tendon healing remains uncertain. One possibility is that they become differentiated into tenocytes within the healing tendon environment and participate in healing through collagen production and remodeling. Alternatively, it has been suggested that BM-MNCs may contribute to healing by acting as “growth factors pumps” rather than through terminal differentiation [11–17].

Chong et al. studied in 57 rabbits the effect of inoculation of BM-MNCs in an Achilles tendon injury model. A transection in the Achilles tendon was performed and either treated with Kessler suture with or without the addition of MSCs. Histological, immunohistochemistry, morphometric, and mechanical testing was performed. The BM-MNCs improved mechanical and histological parameters only at early stages (3 weeks), suggesting the effect on accelerating healing at early time period [8]. Multiple questions still remain uncertain. Time is crucial for biological therapies. It is not clear at what time point the inoculation should be considered and the number of applications needed. Should be wait for 6 months of nonresponding medical treatment or try this therapy earlier? Should be inoculate these cells alone or combine them with growth factors such as PRP? Shall we give only one injection or try serial inoculations? In all our patients, we did one inoculation of BM-MSC after 6 months of failed nonoperative treatment. However, in the one patient that did not improve, probably a second inoculation of BM-MNCs or inoculation of PRP would may have been the answer.

We believe that this therapy could be considered as an alternative treatment for those patients who have failed nonoperative treatment before surgical intervention is considered. Further control studies will be needed to determine if inoculation of BM-MNCs can improve tendon healing in patients with chronic patellar tendinopathy. According to this study, inoculation of BM-MNCs for the treatment of patellar tendinopathy is a promising therapeutic approach.

## 5. Conclusions

This study investigates the use of BM-MNCs for the treatment of chronic patellar tendinopathy. Patients showed statistically clinical improvement at 5-year followup. Inoculation of BM-MNCs could be considered as a potential therapy for those patients with chronic patellar tendinopathy refractory to nonoperative treatments.

## Figures and Tables

**Table 1:** Sonographic grading of the patellar tendinopathy. Grade ratio between injured area and whole tendon section at US scan.

Grade	
1	Injured area is less than 20% of the whole tendon section
2	Injured area is between 20% and 50% of the whole tendon section
3	Injured area is more than 50% of the whole tendon section
4	Subtotal or total tear, respectively, with partial and total tendon retraction

**Table 2:** Outcome score changes from preoperative results to final results, with *P* value for all outcome scales.

Knee scoring system	Preoperative	Postoperative	*P* value
Tegner	2	8	0.0061
Lysholm	33	53	0.1043
IKDC	36	69	0.047
KOOS			
Pain	47	63	0.2399
Symptoms	44	71	0.0086
ADL	63	90	0.0246
Sport	24	63	0.0078
QOL	50	71	0.0825
SF-12			
Mental	52	57	0.5589
Physical	41	44	0.438

IKDC: international knee documentation Committee; KOOS: knee injury and osteoarthritis outcome score; ADL: activities of daily living; QOL: quality of life; SF-12: short form-12.
